# Variation in *MSRA* Modifies Risk of Neonatal Intestinal Obstruction in Cystic Fibrosis

**DOI:** 10.1371/journal.pgen.1002580

**Published:** 2012-03-15

**Authors:** Lindsay B. Henderson, Vishal K. Doshi, Scott M. Blackman, Kathleen M. Naughton, Rhonda G. Pace, Jackob Moskovitz, Michael R. Knowles, Peter R. Durie, Mitchell L. Drumm, Garry R. Cutting

**Affiliations:** 1 McKusick-Nathans Institute of Genetic Medicine, Johns Hopkins University School of Medicine, Baltimore, Maryland, United States of America; 2 Division of Pediatric Endocrinology, Johns Hopkins University School of Medicine, Baltimore, Maryland, United States of America; 3 Cystic Fibrosis/Pulmonary Research and Treatment Center, School of Medicine, The University of North Carolina at Chapel Hill, Chapel Hill, North Carolina, United States of America; 4 Department of Pharmacology and Toxicology, University of Kansas, Lawrence, Kansas, United States of America; 5 Division of Gastroenterology, Hepatology, and Nutrition, Hospital for Sick Children, Toronto, Canada; 6 Department of Pediatrics, University of Toronto, Toronto, Canada; 7 Departments of Pediatrics and Genetics, Case Western Reserve University, Cleveland, Ohio, United States of America; University of Washington, United States of America

## Abstract

Meconium ileus (MI), a life-threatening intestinal obstruction due to meconium with abnormal protein content, occurs in approximately 15 percent of neonates with cystic fibrosis (CF). Analysis of twins with CF demonstrates that MI is a highly heritable trait, indicating that genetic modifiers are largely responsible for this complication. Here, we performed regional family-based association analysis of a locus that had previously been linked to MI and found that SNP haplotypes 5′ to and within the *MSRA* gene were associated with MI (*P* = 1.99×10^−5^ to 1.08×10^−6^; Bonferroni *P* = 0.057 to 3.1×10^−3^). The haplotype with the lowest *P* value showed association with MI in an independent sample of 1,335 unrelated CF patients (OR = 0.72, 95% CI [0.53–0.98], *P* = 0.04). Intestinal obstruction at the time of weaning was decreased in CF mice with *Msra* null alleles compared to those with wild-type *Msra* resulting in significant improvement in survival (*P* = 1.2×10^−4^). Similar levels of goblet cell hyperplasia were observed in the ilea of the *Cftr* ^−/−^ and *Cftr* ^−/−^ *Msra* ^−/−^ mice. Modulation of MSRA, an antioxidant shown to preserve the activity of enzymes, may influence proteolysis in the developing intestine of the CF fetus, thereby altering the incidence of obstruction in the newborn period. Identification of *MSRA* as a modifier of MI provides new insight into the biologic mechanism of neonatal intestinal obstruction caused by loss of CFTR function.

## Author Summary

Cystic fibrosis (CF) is a monogenic disease with considerable phenotypic variability. About 15% of newborns with CF suffer from an intestinal obstruction called meconium ileus (MI), and studies in CF twins have shown that modifier genes play a substantial role in the development of this complication. We used a family-based study design to enrich for genetic modifiers of MI and found that variations in the *MSRA* gene, represented by combinations of SNPs, or haplotypes, were protective against this manifestation of CF. We investigated association between one of the *MSRA* haplotypes and MI in an independent sample of CF patients and showed that it had a similar protective effect. Furthermore, CF mice lacking *Msra* expression had lower mortality due to intestinal obstruction at the time of transitioning to solid food and lived longer than CF mice with normal *Msra*, thus supporting the protective effect of the haplotype we observed in human CF subjects. The identification of modifiers of MI such as *MSRA* offers new insight into the mechanism of this life-threatening complication of CF.

## Introduction

Cystic fibrosis (CF; MIM 219700, http://www.omim.org) is an autosomal recessive condition caused by mutations in the cystic fibrosis transmembrane conductance regulator (CFTR; MIM 602421) gene [1]. The earliest manifestation of CF is meconium ileus (MI), a prenatal obstruction of the small intestine at the ileocecal junction. Meconium, the intestinal contents of the developing gut that form the first bowel movement, has an abnormally high protein content in CF neonates thought to be due to defective proteolysis [2]–[4]. Impaction of the tenacious meconium results in intestinal obstruction in approximately 15% of CF newborns. This complication presents as abdominal distention, failure to pass meconium, and vomiting and was near universally fatal in CF newborns until effective treatment (enema or surgery) was developed. The long term effects of MI have been a matter of debate as some investigators have reported worse outcomes while others observed no significant differences from CF subjects without MI [5]–[7].

Genetic modifiers have been implicated in the development of MI for over 40 years as recurrence risk for this complication in siblings with CF (∼0.25) has consistently been shown to be higher than that in unrelated individuals with CF (∼0.15) [8]–[12]. Concordance analysis in monozygous and dizygous twins demonstrated that the heritability of MI approaches 1.0, confirming that modifier genes play a substantial role in MI [12]. *CFTR* also contributes to risk as MI almost exclusively manifests in CF subjects with exocrine pancreatic insufficiency (PI), which is highly correlated with *CFTR* genotype [13], [14]. Furthermore, the incidence of MI appears to vary among *CFTR* mutations that confer PI. For example, the amino acid substitution p.Gly551Asp (“G551D”) has been associated with reduced risk of MI compared to the most common *CFTR* mutation that causes a deleterious in-frame deletion of one amino acid (p.Phe508del, “delta F508”) [15], [16].

Initial attempts to identify MI modifier genes in humans utilized localization results from mouse studies. Mice with disruption of *Cftr* present with an MI-like phenotype; however, it differs from human MI in several respects. First, intestinal obstruction in CF mice causes mortality shortly after birth and at the time of weaning with the introduction of solid food [17]–[19]. Life-threatening obstruction in humans occurs in the perinatal period, while episodes of variable severity termed distal intestinal obstruction syndrome (DIOS) can also occur throughout life (5 to 12 episodes per 1,000 patient-years), especially in adults with PI and as a post-operative complication of surgical intervention, particularly organ transplantation [20], [21]. Second, obstructive lesions in CF mice have been observed in the jejunum, ileum and colon, compared to predominantly ileo-colic localization in humans [22]. Third, pancreatic exocrine disease is much less prominent in mouse models of CF [17], [23], [24]. On the other hand, there are instructive genetic similarities between mice and humans with CF. *Cftr* alleles influence the rate and severity of murine intestinal obstruction [17], [25]–[28] and strain-specific differences in the penetrance of intestinal obstruction indicate that different modifier genes underlie obstruction at birth and at weaning [19]. Candidate gene approaches in CF mice have revealed that decreased expression of the sodium hydrogen exchanger 3 (*Nhe3*) or mucin 1 (*Muc1*) or over-expression of the chloride calcium channel activated 3 protein (*Clca3/Gob5*) can reduce intestinal obstruction at weaning [29]–[31].

Newer animal models of CF have provided additional clues in the search for modifiers of MI. *CFTR* knock-out ferrets and pigs have been shown to develop intestinal obstruction that is anatomically and temporally equivalent to that observed in humans; however, the phenotype is highly penetrant in these animals (75% and 100%, respectively) [32], [33]. Animal models of CF and heritability studies in humans suggest that intestinal obstruction due to loss of CFTR function is a consistent feature, and that the incomplete penetrance of this trait in humans with CF is likely due to the presence of genetic modifiers. We present here the results of a regional association analysis of a linked locus on chromosome 8 [12], and report the identification and functional confirmation of a modifier gene for MI in humans and mice with CF.

## Results

### Regional association analysis of CF patients implicates variants near and within *MSRA* as modifiers of MI

**Figure 1:**
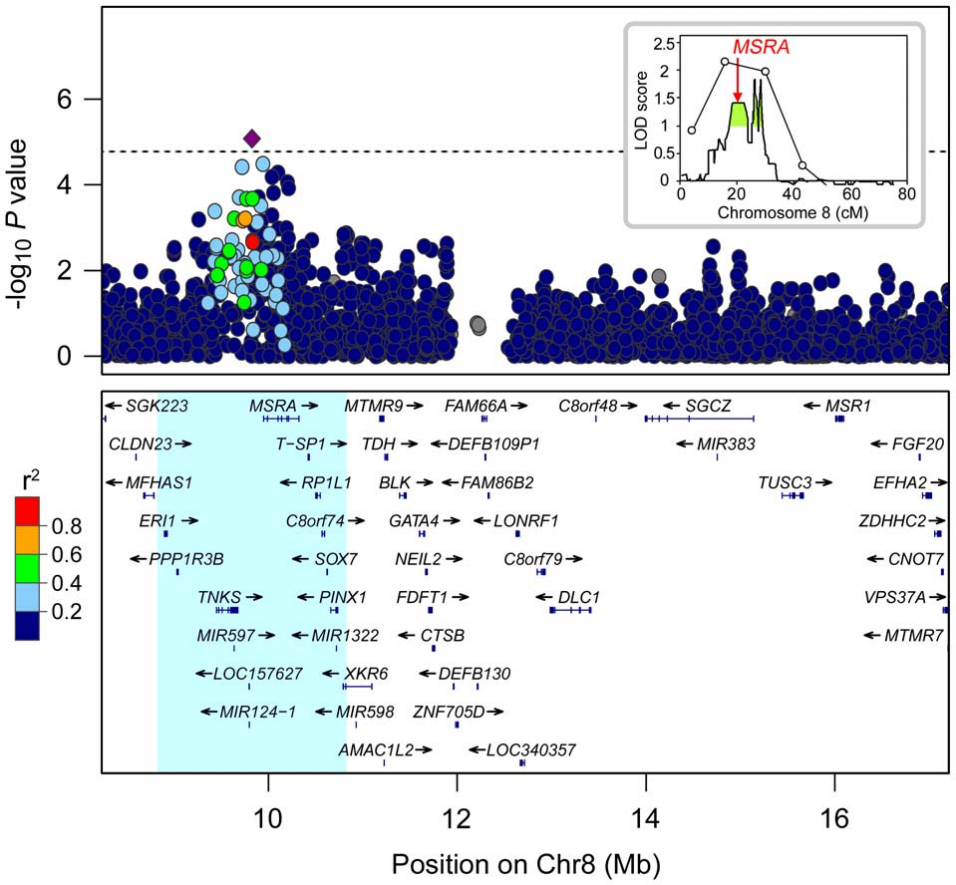
Regional association of SNPs within a region of linkage to MI on chromosome 8p23.1. The inset plot shows the locus linked to MI on chromosome 8 identified by Blackman, et al [12]. The green shaded region under the peak extending from 17.9 cM (8.2 Mb) to 29.0 cM (17.2 Mb), where LOD score >1, indicates the region analyzed in the main plot. The map position of *MSRA* is denoted by an arrow at ∼20.2 cM. In the main plot, *P* values are plotted in log scale versus physical location in Mb. The SNP showing the strongest association with MI, rs614197, is represented by a diamond (*P* = 8.35×10^−6^). SNPs surrounding rs614197 are color coded to reflect their LD with this SNP (pair-wise r^2^ using 1000 Genomes CEU, August 2009). The dashed line indicates the threshold for region-wide significance after Bonferroni correction for 2,896 SNPs (P<1.73×10^−5^). Genes, exon positions, and direction of transcription are denoted below plot (human genome build 18). Nine genes outside this interval were omitted for display purposes: *C8orf12*, *FAM167A*, *DEFB136*, *DEFB135*, *DEFB134*, *FAM66D*, *LOC392196*, *USP17L2*, and *FAM86B1*. The blue shaded region represents the 2 Mb encompassing rs614197 in which haplotype association was tested (see Figure S1).

To investigate a region on chromosome 8 that had previously shown linkage to MI [12], transmission analysis of SNPs was performed using families from the Cystic Fibrosis Twin and Sibling Study (TSS). As MI is associated with an increased recurrence risk among siblings, we enriched for genes that modify this phenotype by analyzing 133 families in which at least one subject was affected with MI (26 MI concordant and 91 discordant pairs, 2 concordant and 8 discordant sets of 3, and 6 singletons; Table 1). Since MI rarely occurs in the absence of PI, individuals were excluded if their *CFTR* genotypes were associated with exocrine pancreatic sufficiency (PS) (n = 7) or if they clinically demonstrated PS (n = 5). All SNPs on the Illumina 610-Quad genotyping panel that passed rigorous quality control (2,896 SNPs) [34] were included from an approximately 9 Mb region within the linkage locus where the SNP LOD score exceeded 1.0 (boundaries: rs2945913-rs2285274; green shaded area in linkage plot inset, Figure 1). Parental genotypes were utilized to test the transmission of SNP alleles using pedigree-based association testing (PBAT), an extension of the transmission disequilibrium test that is robust against population stratification [35], [36]. A cluster of SNPs proximal to and within *MSRA* (MIM 601250) showed evidence of association with MI when an additive mode of inheritance was assumed (Figure 1). One SNP within this cluster 5′ to *MSRA*, rs614197, exceeded the threshold for region-wide significance (*P* = 8.35×10^−6^, Bonferroni corrected *P* = 0.024). However, this SNP was not associated with MI in a sample of unrelated CF patients from the Canadian Consortium for Genetic Studies (CGS; Table 1).

**Table 1:** Patient characteristics.

	CF Twin and Sibling Study (TSS)			Canadian Consortium for Genetic Studies (CGS)	
	MI	No MI (Families)^a^	No MI (Entire Sample)^b^	MI	No MI
Subjects	166	104	766	220	1,163
Mean Age in Years ± SD	16.3±6.8	16.7±6.7	18.9±9.5	23.1±8.7	26.3±10.6
Females (%)	82 (49.4)	52 (50.0)	358 (46.7)	108 (49.1)	532 (45.7)
p.Phe508del Homozygotes (%)	104 (62.7)	61 (58.7)	402 (52.5)	152 (69.1)	699 (60.1)

**^a^**Primary analysis included subjects from 133 “MI families” in which at least one sibling had MI.

**^b^** *CFTR* mutation-specific analysis (i.e. p.Gly551Asp vs. p.Phe508del) utilized the entire TSS sample.

**Figure 2:**
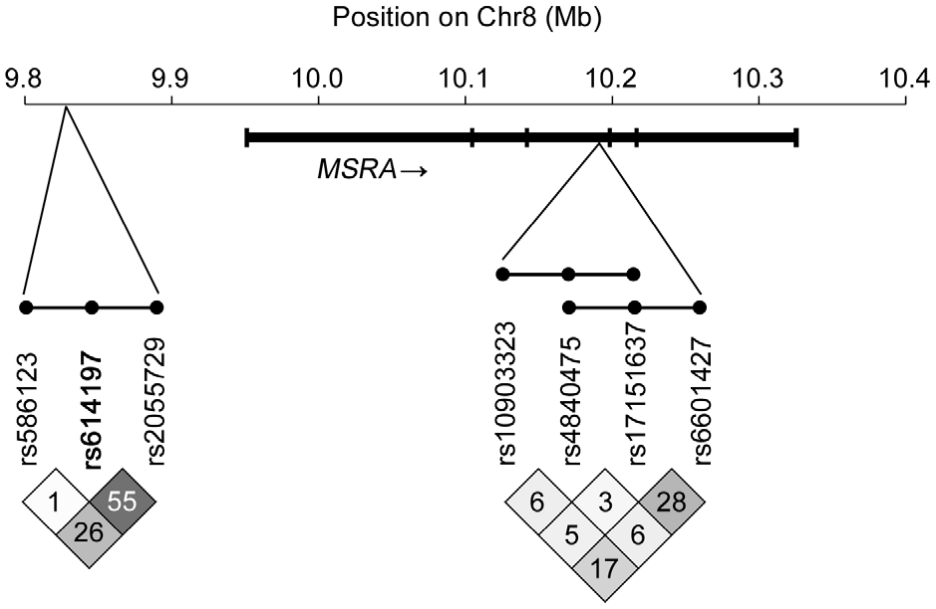
Haplotype association in *MSRA* region. In this schematic, haplotypes comprised of three consecutive SNPs are represented as dots connected by a line. Two overlapping haplotypes localized within intron 3 of *MSRA* had a statistically significant protective effect on MI (*P*<1.73×10^−5^ after Bonferroni correction for 2,890 haplotypes tested in a 2 Mb region): rs10903323 T – rs4840475 G – rs17151637 A and rs4840475 G – rs17151637 A – rs6601427 C. A “risk” haplotype upstream of *MSRA*, rs586123 G – rs614197 G – rs2055729 C, was just below the threshold for significant association. Linkage disequilibrium patterns (pairwise r^2^) are displayed below the rs numbers corresponding to each SNP (physical location provided in Table 2).

Lack of significant association between MI and rs614197 in the CGS sample led us to question whether the initial observation in the TSS families was spurious, or if detection of association could be confounded by interacting loci [37] or by heterogeneity of effect of alleles at the locus [38], [39]. To test the latter concept, we used haplotype analysis to search for additional genetic variation associated with MI in the region surrounding *MSRA*. Haplotypes derived from a sliding window of three consecutive SNPs across a 2 Mb region centered at rs614197 (boundaries: rs17700611-rs4240673) were tested for transmission distortion under an additive genetic model (Figure S1). We used combinations of three SNPs as a compromise between the number of haplotypes generated and the penalty incurred by multiple testing. Two haplotypes exceeded the threshold for significant regional association after Bonferroni correction for 2,890 different haplotypes observed in the 133 families studied (Table 2). The combination of rs10903323 T, rs4840475 G and rs17151637 A (T-G-A) spanning a 3.5 kb region in intron 3 of *MSRA* (Figure 2) was significantly over-transmitted to individuals *without* MI, or in other words had a protective effect on MI (54 informative families, raw *P* = 1.08×10^−6^; Bonferroni *P* = 3.13×10^−3^). In the entire TSS cohort, the T-G-A haplotype frequency was 14.9% (13.0% in subjects with MI, 25.0% in those without MI). A second, overlapping haplotype (Figure 2) containing two of the same SNP alleles as the T-G-A haplotype (rs4840475 G, rs17151637 A, plus rs6601427 C; Table 2) similarly demonstrated association with the absence of MI (48 informative families, raw *P* = 1.17×10^−5^; Bonferroni *P* = 0.034). Another haplotype located just 5′ to *MSRA* showed nearly significant over-transmission to individuals *with* MI, indicating that it conferred *risk* for MI (59 informative families, raw *P* = 1.99×10^−5^; Bonferroni *P* = 0.057). Interestingly, this haplotype included rs614197, the SNP that was associated with MI in the initial single marker analysis (rs586123 G, rs614197 G and rs2055729 C; Table 2). The SNPs comprising these three haplotypes displayed weak linkage disequilibrium (LD) with the exception of the second and third SNP in the 5′ haplotype (r^2^ = 0.55; Figure 2). Thus, the haplotypes are likely detecting additional genetic variation that cannot be assessed by the tagging SNPs on this array platform.

**Table 2:** Key haplotypes in *MSRA* region showing association with MI in TSS cohort.

SNP 1	SNP 2	SNP 3	Haplotype^b^	Frequency	*P* value
rs10903323 (10186977)^a^	rs4840475 (10190009)	rs17151637 (10190492)			
**T**	**G**	**A**	**T-G-A**	**0.149**	**1.08×10^−6^** *
T	G	G	T-G-G	0.428	3.10×10^−3^
T	A	A	T-A-A	0.101	3.23×10^−1^
T	A	G	T-A-G	0.189	6.71×10^−1^
C	G	G	C-G-G	0.124	7.34×10^−1^
rs4840475 (10190009)	rs17151637 (10190492)	rs6601427 (10193435)			
**G**	**A**	**C**	**G-A-C**	**0.144**	**1.17×10^−5^** *
G	G	C	G-G-C	0.290	6.48×10^−2^
G	G	T	G-G-T	0.264	1.47×10^−1^
A	A	C	A-A-C	0.095	2.21×10^−1^
A	G	T	A-G-T	0.177	9.08×10^−1^
rs586123 (9823904)	*rs614197* (9825992)	rs2055729 (9830072)			
**G**	**G**	**C**	**G-G-C**	**0.186**	**1.99×10^−5^** ‡
G	A	T	G-A-T	0.629	7.07×10^−4^
G	A	C	G-A-C	0.039	9.69×10^−2^
A	*A*	C	A-A-C	0.099	3.17×10^−1^
G	G	T	G-G-T	0.022	6.70×10^−1^

**^a^**Numbers in parentheses indicate physical position on chr8 in base pairs (NCBI Build 36).

**^b^**All observed haplotypes with frequency >1% are shown; bold indicates haplotypes noted in text.

*Protective haplotypes that reached statistical significance after Bonferroni correction for all 2,890 haplotypes tested (*P*<1.73×10^−5^).

**‡:** Near significant risk haplotype containing rs614197 (italicized), the SNP that was most highly associated with MI in the initial analysis.

To evaluate whether the *MSRA* alleles identified here by association could explain the former linkage signal on chromosome 8, the frequency of the T-G-A haplotype that showed the strongest association signal was calculated in siblings with CF that were concordant for MI. Sixteen sib-pairs contributed positively to the LOD score while the remaining 14 sib-pairs had a negative LOD score. The frequency of the T-G-A *MSRA* haplotype was lower in siblings that contributed to linkage (5 of 64 chromosomes, 7.8%) compared to siblings that did not (12 of 44 chromosomes, 21.4%; *P* = 0.033). Given that the T-G-A haplotype is associated with a *decreased* rate of MI, it appears that the observed linkage with risk for MI in our previous study was due to the increased sharing of *other MSRA* haplotypes that confer a higher risk of MI.

A replication study was performed using CF patients recruited by the CGS. The CGS is representative of the general CF population in Canada and comprises approximately 70% of all Canadian CF patients [40]. The overall rate of MI in this group was 15.9% (n = 220 with MI, 1,163 without MI), which is consistent with the incidence of MI reported in numerous Caucasian CF populations [8], [10], [16]. The frequency of the T-G-A haplotype in the 1,335 CGS subjects in whom haplotypes were ascertained was similar (15.4%) to that observed in the TSS (14.9%; Table 2). The T-G-A haplotype showed association with MI in the CGS sample under an additive model (OR = 0.72, 95% CI [0.53–0.98], *P* = 0.04), in agreement with the initial finding in the TSS. The incidence of MI in subjects with 0 copies of the T-G-A haplotype was 17.3% (165/954 subjects), 13.1% (46/351) in those with 1 copy and 10% (3/30) in those with 2 copies.

The concept that variation in *CFTR* influences the risk of MI is evident from the observation that a PI state (primarily determined by *CFTR* genotype) is required for the development of MI [14]. However, there is a finer correlation between *CFTR* genotype and MI as two *CFTR* mutations that are highly correlated with PI, p.Gly551Asp and p.Gly542X, have been shown to decrease or increase MI risk, respectively, from that conferred by the common mutation p.Phe508del [14]–[16]. As p.Gly551Asp is present at a relatively high frequency among European CF alleles (∼2% [41]), we evaluated the association between this mutation and MI in the TSS and CGS cohorts. The incidence of MI in p.Gly551Asp-bearing subjects was 7.8% in the TSS (n = 51) and 5.9% in the CGS (n = 51), compared to 20.5% (n = 507) and 17.9% (n = 851) in p.Phe508del homozygotes (*P* = 0.026, 0.033), respectively. Combining these two samples of CF subjects demonstrated that the odds of MI in subjects with p.Gly551Asp was about a third of that in p.Phe508del homozygotes (OR = 0.32, 95% CI [0.13–0.76]; *P* = 0.010), comparable to the report by Hamosh, *et al* [15]. The finding that variation in the disease-causing gene alters the incidence of MI even among PI subjects suggested that the relationship between MI and the *MSRA* haplotype could be confounded by variation in *CFTR*. To control for genetic heterogeneity in *CFTR*, we tested for association between the T-G-A haplotype and MI by evaluating 1,017 subjects with the same genotype (p.Phe508del homozygotes) drawn from the TSS MI families (n = 166) and CGS studies (n = 851). A meta-analysis conducted using logistic regression coefficients and standard errors [42] from individual TSS and CGS analyses revealed that the T-G-A haplotype retained an additive protective effect (*P* = 0.001), indicating that *MSRA* modifies MI independently of variation in *CFTR*.

### *Msra* modifies intestinal obstruction and survival in CF mice

**Figure 3:**
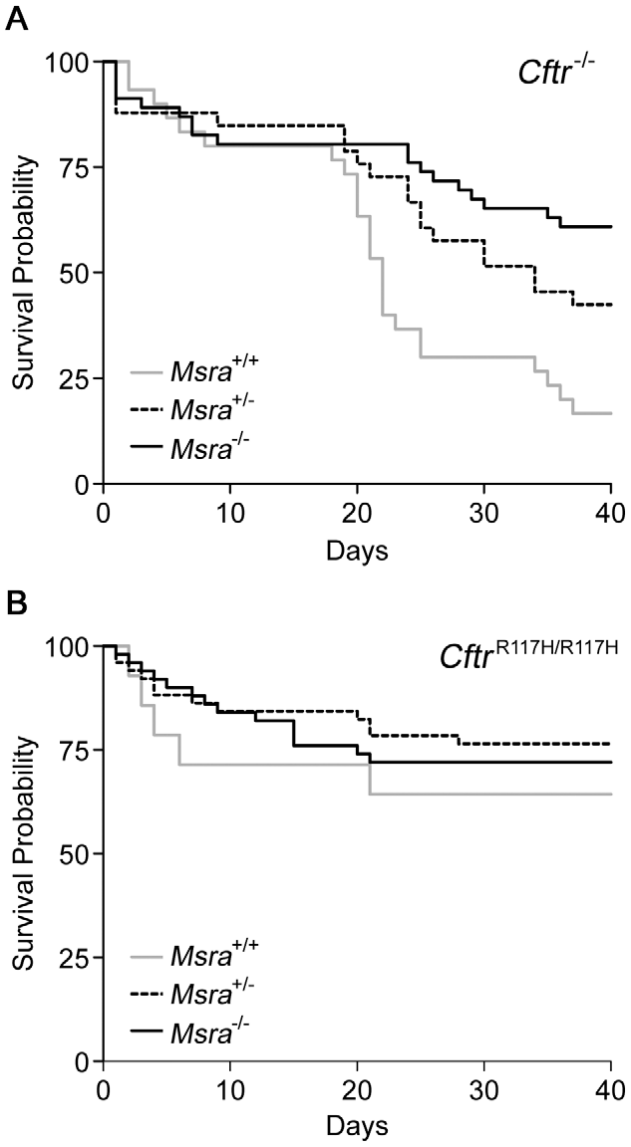
Kaplan-Meier survival curves in CF mice according to *Msra* genotype. A. CF mice homozygous for a null *Cftr* allele (*Cftr* ^−/−^) and wild-type for *Msra* show high mortality due to intestinal obstruction around the time of weaning (ca. 21 days; n = 30). In contrast, survival is markedly improved in *Cftr* ^−/−^ mice lacking one (n = 33) or two *Msra* alleles (n = 46) compared to wild-type (*P* = 0.022 and *P* = 0.0001, respectively; log-rank test). B. CF mice homozygous for a missense *Cftr* allele (*Cftr* ^R117H/R117H^) and wild-type for *Msra* display a low rate of mortality due to intestinal obstruction around the time of weaning (n = 14). Survival is not affected in *Cftr* ^R117H/R117H^ mice lacking one (n = 51) or two (n = 51) *Msra* alleles compared to wild-type.

Intestinal obstruction at the time of weaning is the primary cause of death in mouse models of CF [22], [43]. As the effect of the MI-associated haplotypes upon MSRA expression was unknown, we elected to introduce null alleles of *Msra* into mouse models of CF with high and low rates of mortality due to intestinal obstruction to detect whether loss of *Msra* expression reduced or exacerbated the rate of obstruction. Intestinal obstruction in a null CF mouse model (C57BL/6J *Cftr* ^−/−^) leads to high mortality (>80% [17]) by 40 days of age while lower rates of mortality [44] occur in a CF mouse model (C57BL/6J^R117H/R117H^) with a targeted knock-in of a missense mutation (p.Arg117His) associated with residual CFTR function [45], [46] and very low rates of MI in humans with CF [47]. Mice heterozygous for the *Cftr* null (*Cftr* ^+/−^) or p.Arg117His allele (*Cftr* ^+/R117H^) were crossed to mice with one or two *Msra* null alleles to produce CF mice (*Cftr* ^−/−^ or *Cftr* ^R117H/R117H^) with wild-type (+/+), heterozygous (+/−), and null (−/−) *Msra* genotypes. As expected, *Cftr* ^−/−^ mice displayed a sharp drop in survival around the time of weaning when solid food is introduced to the diet (Figure 3). The median survival of the *Cftr* ^−/−^ mice was 22 days, consistent with the high rates of mortality due to intestinal obstruction reported in other CF ‘null’ mice [17], [18], [24], [48], all of which would have been homozygous for wild-type *Msra* (i.e. *Msra* ^+/+^). However, survival was significantly improved in *Msra* ^+/−^ and *Msra* ^−/−^ CF mice compared to their *Msra* ^+/+^ littermates (*P* = 0.022 and *P* = 1.2×10^−4^, respectively, by log-rank test; Figure 3A). At the end of the follow-up period, 61% of *Msra* ^−/−^ and 42% of *Msra* ^+/−^ mice were still living compared to 17% of *Msra* ^+/+^ mice. The increasing trend in survival across genotypes (*P*_trend_ = 1.3×10^−4^) mirrors the additive effect of the *MSRA* haplotype observed in humans. As anticipated, *Cftr* ^R117H/R117H^ mice displayed reduced mortality, notably through the weaning period, compared to *Cftr*-null mice (64.3% of *Msra* ^+/+^ mice alive at 40 days, n = 14; Figure 3B). However, there was no difference in the rate of survival between *Msra* ^+/+^ mice and mice with one *Msra* null allele (*Msra* ^+/−^: 76.5% alive at 40 days, n = 51; *P* = 0.33, log-rank test) or two null alleles (*Msra* ^−/−^: 70.6% alive at 40 days, n = 51; *P* = 0.51). Mortality due to intestinal obstruction was confirmed in all animals for which the carcass was identified intact, and these were primarily animals that succumbed after weaning. Thus, loss of *Msra* expression increased survival in *Cftr* ^−/−^ mice by reducing the rate of fatal intestinal obstruction.

**Figure 4:**
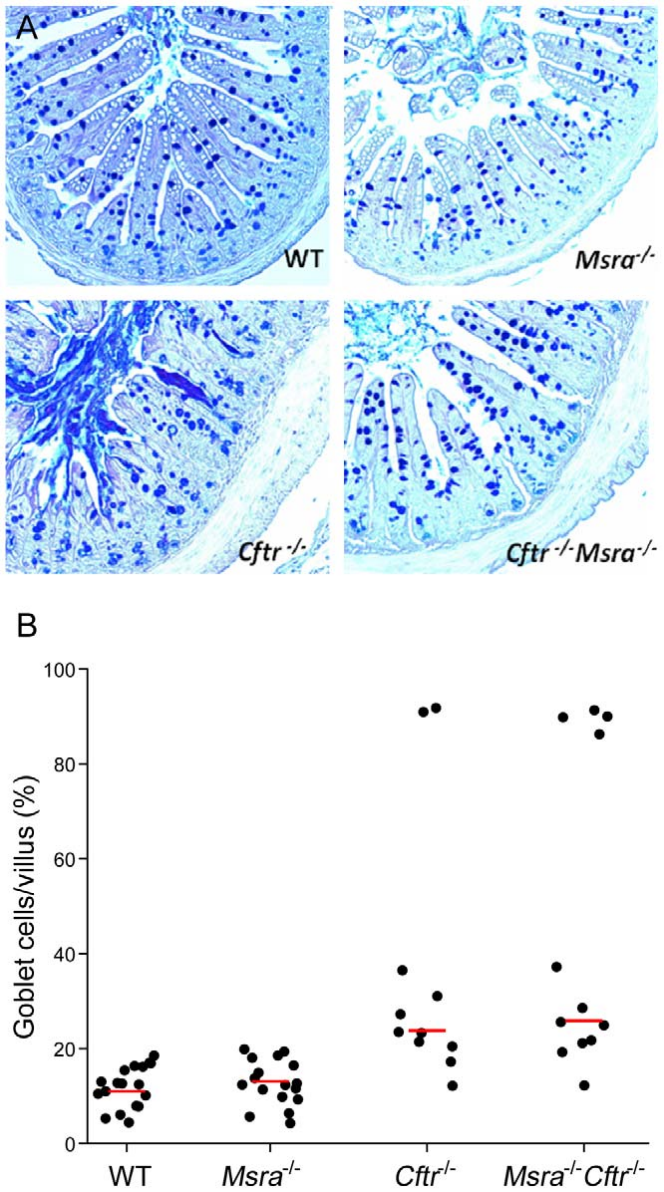
Histology of small intestine and goblet cell fraction of villi in WT, *Cftr* ^−/−^, *Msra* ^−/−^, and *Cftr* ^−/−^ *Msra* ^−/−^ mice. A. PAS/Alcian blue stained sections of ileum from 15 day old mice. Genotypes are shown in the bottom right hand corner of each section. Goblet cells laden with acid mucin are stained blue. The *Cftr* ^−/−^ section demonstrates mucus accumulation in the lumen and crypts with blunted and necrotic villi. B. Percentage of goblet cells per villus epithelial cells in the ileum of 15 day old mice. Each dot represents one section. Median goblet cell densities (indicated by red lines) were 11.1% in WT (n = 4 mice; 10,919 cells counted in 18 sections), 13.0% in *Msra* ^−/−^ (n = 3 mice; 9,586 cells counted in 17 sections), 23.7% in *Cftr* ^−/−^ (n = 2 mice; 4,762 cells counted in 11 sections), and 25.9% in *Cftr* ^−/−^ *Msra* ^−/−^mice (n = 3 mice; 6,396 cells counted in 12 sections).

Excessive mucus accumulation in the crypts and lumen along with goblet cell hyperplasia are characteristic findings in the small and large intestine of *Cftr* ^−/−^ mice [17], [22], [43]. As goblet cells are the primary source for mucus in the intestine, we sought to determine if the goblet cell content of villi observed in the ileum of 15 day old mice is affected by *Msra* expression (Figure 4A). In wild-type (WT; i.e. non-CF) mice, goblet cell counts per villus ranged from 7.6% to 19.7% with a median of 11.1% (Figure 4B). The fraction of goblet cells in *Msra* ^−/−^ mice was similar to WT, ranging from 5.8% to 19.9% with a median of 13.0%, while the fraction of goblet cells in *Cftr* ^−/−^ mice had a wider range, 12.4% to 90.1%, and higher median (23.7%) than WT or *Msra* ^−/−^ mice (Figure 4B). The fraction of goblet cells in WT and *Msra* ^−/−^ mice and the increased proportion in *Cftr* ^−/−^ mice is comparable to the numbers reported in other studies [29], [31]. Like the *Cftr* ^−/−^ mice, goblet cell fraction in the ileum of *Cftr* ^−/−^ *Msra* ^−/−^ mice varied widely, ranging from 16.3% to 90.1% with a median of 25.9% (Figure 4B). *Cftr* ^−/−^ and *Cftr* ^−/−^ *Msra* ^−/−^ mice displayed considerable heterogeneity in the fraction of goblet cells per section with both groups having a subset of villi where ∼90% of cells were goblet cells (two sections in *Cftr* ^−/−^ and four in *Cftr* ^−/−^ *Msra* ^−/−^).

As goblet cell fractions were not normally distributed for the *Cftr* ^−/−^ and *Cftr* ^−/−^ *Msra* ^−/−^ mice, we evaluated these differences using the non-parametric Mann-Whitney test. WT and *Msra* ^−/−^ mice had similar distributions (*P* = 0.97) whereas both differed significantly from *Cftr* ^−/−^ (WT: *P* = 7.6×10^−5^; *Msra* ^−/−^: *P* = 2.6×10^−4^). Similarly, *Cftr* ^−/−^ *Msra* ^−/−^ mice differed from WT (*P* = 2.4×10^−5^) and *Msra* ^−/−^ mice (*P* = 3.8×10^−5^). However, the distributions in *Cftr* ^−/−^ and *Cftr* ^−/−^ *Msra* ^−/−^ did not differ when all observations were included (*P* = 0.67) or when the sections with goblet cell fractions exceeding 89% were excluded (median 22.2% vs. 22.3%, respectively; *P* = 0.26). Thus, loss of *Msra* expression does not appear to affect goblet cell hyperplasia in the ileum of CF mice despite reducing intestinal obstruction and increasing survival.

## Discussion

Neonatal intestinal obstruction (also known as meconium ileus or MI) has incomplete penetrance (15%) and high heritability (∼1.0) suggesting a prominent role for modifier genes in this complication of CF [12]. Both candidate gene and genome-wide studies indicate that multiple genetic modifiers of low effect contribute to this trait in humans and in mice [12], [19], [49], [50]. The polygenic etiology of MI combined with its low incidence in CF present a substantial challenge to identifying the responsible genetic modifiers. However, by employing both linkage and transmission methods in a family-based study followed by replication in an unrelated sample of CF patients, we were able to implicate the *MSRA* gene on chromosome 8. To test whether manipulation of *Msra* expression modified intestinal obstruction in the CF mouse, we elected to use a null allele of *Msra* to avoid temporal or spatial issues that might have complicated a transgenic over-expression strategy. As we did not know if loss of expression would increase or decrease the rate of obstruction, we employed two CF mouse models with different rates of mortality due to intestinal obstruction at the time of weaning. Our hypothesis was that the CF null model (with high rates of obstruction) would reveal whether loss of Msra function *decreased* obstruction while the p.Arg117His model (with lower rates of obstruction) would reveal whether loss of Msra function *increased* obstruction. Indeed, reduction of *Msra* expression in the null CF mouse model resulted in a significant decrease in intestinal obstruction. The lack of effect in the p.Arg117His CF mice suggested that the modifying effect of *Msra* did not exceed the reduction in obstruction conferred by residual function of CFTR bearing p.Arg117His. Together, the two CF mouse models indicated that loss of *Msra* afforded protection from intestinal obstruction during the time of weaning.

The *Msra* null allele was generated from 129-derived embryonic stem cells, and mortality from obstruction is nearly 100% in mice on the 129 background [17]. Thus, it is unlikely that variation in the 129-derived region surrounding *Msra* is responsible for the reduced intestinal obstruction. Furthermore, the *Msra* region in mice displays minimal synteny with the region surrounding *MSRA* on chromosome 8 in humans. For example, tankyrase (*TNKS*), a gene adjacent to the 5′ end of *MSRA* in humans is not adjacent to *Msra* on chromosome 14 in the mouse but is located on mouse chromosome 8. The mouse studies provide compelling evidence supporting the contention that *MSRA* modulates MI in humans. Interestingly, non-CF *Msra* ^+/−^ mice have previously been shown to have *reduced* lifespan thought to be the consequence of enhanced vulnerability to oxidative stress compared to wild-type animals [51]. In contrast, we observed longer survival in CF mice lacking *Msra*. Our opposing finding suggests that in the disease context of CF, having *less* Msra affords a protective benefit.

The initial starting point in our modifier gene search was a 9 Mb region on chromosome 8 within a region linked to risk for MI in 30 concordant sibling pairs [12]. Association analysis of 133 families using transmission disequilibrium testing identified a single SNP (rs614197) that achieved region-wide significance. Lack of significant association between MI and this SNP in the CGS sample motivated us to search for evidence of untyped alleles using haplotype analysis. The association of both ‘protective’ and ‘risk’ haplotypes with MI led us to surmise that alleles of different effect exist in or near *MSRA*. We then tested whether the ‘protective’ *MSRA* haplotype had a similar influence on MI in an independent CF sample. While the TSS sample was potentially subject to ascertainment bias due to its recruitment criteria (i.e. having a sibling with CF), the CGS sample was ideal for replication as recruitment was based only on a diagnosis of CF, and the sample comprises 70% of the patients with CF in Canada. The significant correlation between the ‘protective’ haplotype and reduced incidence of MI in the CGS sample and conformity with an additive model provided reassurance that variants in *MSRA* modified MI risk. Finally, as several studies, including the present study, have indicated that the *CFTR* genotype can affect the rate of MI [14]–[16], we evaluated whether *CFTR* allelic variation contributed to the association between the *MSRA* haplotype and MI. The T-G-A haplotype associated with MI in an additive fashion in individuals with identical *CFTR* genotypes (p.Phe508del homozygotes), thereby demonstrating that *MSRA* modifies MI independently of variation in *CFTR*, the disease-causing gene.

Heterogeneity of effect appears to explain the observed linkage of *risk* for MI to the region encompassing *MSRA*. As noted above, the T-G-A haplotype demonstrating robust association with MI conferred *protection* from MI rather than *risk* for MI. However, this is not the most common haplotype (∼15%); thus most siblings carry other haplotypes derived from the alleles of the three SNPs that, by definition, confer higher risk for MI than the protective T-G-A haplotype. As predicted by this assumption, the T-G-A haplotype was significantly underrepresented in siblings concordant for MI who contributed to the linkage signal on chromosome 8. By the same token, the T-G-A haplotype was *over* represented in siblings who did not contribute to linkage. Hence, the observed modest linkage in siblings concordant for MI was the result of sharing of neutral *MSRA* haplotypes or haplotypes conferring risk for MI, and lack of sharing of alleles associated with protection from MI. Sequencing of the coding regions of *MSRA* in three CF subjects with MI and no T-G-A haplotypes and in three subjects without MI and two T-G-A haplotypes did not identify any plausible causative variants (data not shown). Thus, we conclude that the haplotypes are tagging as yet unidentified genetic variation within or near *MSRA*.

Central roles for luminal hydration and mucus production in intestinal obstruction in CF mice at the time of weaning have been supported by the manipulation of expression of two genes. In one study, reduced expression of the sodium hydrogen exchanger (*Nhe3*) led to decreased intestinal sodium absorption, thereby increasing the hydration of luminal contents, alleviating obstruction, and improving survival [29]. Similarly, knock-out of the mucin gene *Muc1* in CF mice improved survival due to reduced intestinal mucus content and less obstruction at the time of weaning [30]. As noted by many others [22], [43] as well as in this study, goblet cell hyperplasia is a consistent histologic feature in the small and large intestines of CF mice. However, the role of goblet cells in intestinal obstruction in CF mice is not clear [43]. Reduced expression of *Nhe3* relieved obstruction and *eliminated* goblet cell hyperplasia [29] while increased expression of the goblet cell marker *Clca3* (*Gob5*) relieved obstruction and *increased* goblet cell hyperplasia in CF mice [31]. Rozmahel and colleagues suggested that *Clca3* may reduce mucin release from goblet cells, given the observation of increased goblet cell size, thereby reducing luminal mucus content and intestinal obstruction in the CF mice [31]. Evidence of association between MI and the p.Ser357Asn variant in *CLCA1*, the human ortholog of the murine *Clca3*, in 682 European CF subjects suggests that this goblet cell marker may also contribute to intestinal obstruction in humans [52]. Thus, alteration of mucus content in the CF intestine, either by reduction in goblet cell numbers or down-regulation of mucus release, appears to affect the rate of intestinal obstruction. Our analysis indicated that loss of *Msra* expression did not affect goblet cell hyperplasia in the *Cftr* ^−/−^ ileum despite mitigating intestinal obstruction; thus the link between *Msra* and intestinal obstruction in the context of CF is not immediately clear.

In humans, loss of CFTR function leads to a combination of impaired pancreatic secretion of proteolytic enzymes and deficient luminal hydration of meconium [53], [54]. Consequently, meconium from CF neonates is abnormally proteinaceous compared to that of normal neonates, and it has been proposed that the altered viscoelastic properties of meconium predispose fetuses and neonates with CF to intestinal obstruction [55]. In young CF mice, pancreatic exocrine function is relatively preserved [17]. However, inadequate hydration and excessive mucus secretion leads to distension of the crypts of Lieberkühn in the ileum and colon and formation of concretions that appear to play a role in obstruction [17], [22], [23], [56]. *MSRA* encodes an antioxidant enzyme that modifies the activity of certain proteins by reducing methionine residues [57]. It is expressed in the intestine and other tissues, particularly the liver, kidney, and brain [58]–[60]. A possible link between MSRA and MI may be that MSRA can modulate the activity of proteolytic enzymes [61]. Maximizing the activity of any residual enzymes produced by the fetal pancreas would likely contribute to the breakdown of intestinal proteins *in utero*, thereby reducing the risk of obstruction. Evidence that MSRA modifies intestinal obstruction provides new opportunities to investigate the above concepts and the mechanisms underlying intestinal obstruction in mice and in humans lacking CFTR.

## Materials and Methods

### Ethics statement

This study was approved by the institutional/ethical review boards of all participating institutions. Written, informed consent or assent was obtained from all subjects before enrollment in the study. Experiments on mice were approved by the Case Western Reserve University Institutional Animal Care and Use Committee.

### Study population and phenotype description

Study subjects were derived from the North American CF Modifier Gene Consortium, which is comprised of three independent collections of CF subjects. Subjects in the discovery sample were part of the Cystic Fibrosis Twin and Sibling Study (TSS) at Johns Hopkins University (n = 1,125 subjects). Enrollment was based on conclusive diagnosis of CF [62]. Methods for isolation of patient DNA [63] and identification of *CFTR* mutations [64], [65] have been previously described. The diagnosis of MI was based on the presence of the following features in the newborn period: lack of passage of stool within 24 hours after birth, evidence of intestinal obstruction on abdominal radiograph (ground-glass appearance of intestine, air-fluid levels, and/or intra-abdominal calcifications), evidence of colonic abnormality (microcolon on radiograph), and treatment for obstruction (enema or surgery). Individuals with clinically defined PS, a *CFTR* mutation associated with PS, or unknown pancreatic status were excluded (n = 143), as was one subject with unknown MI status. The primary analysis was conducted in 133 “MI families” in which at least one sibling was affected with MI (270 subjects, 169 parents). Case/control analyses were restricted to persons of self-reported European descent to minimize the potential for spurious associations due to race-related differences in allele frequencies; whereas the primary family-based transmission analysis, which was robust against population stratification, included an additional 23 individuals of non-European or mixed descent.

Findings from the primary analysis were tested in an independent sample which has been described elsewhere [34]. The replication population consisted of 1,573 CF subjects from the CGS [40]. All subjects were defined as having PI. Exclusion of non-whites yielded 1,383 subjects (including 56 sib-ships) for analysis. Rates of MI in subjects carrying the *CFTR* p.Gly551Asp mutation (c.1652G>A) or who were homozygous for p.Phe508del were evaluated in the entire TSS sample and the CGS sample. Subjects with p.Gly551Asp carried this mutation in *trans* with another PI-associated mutation: p.Cys343X (c.1029delC), c.1585-1G>A, p.Lys1177SerfsX15 (c.3528delC), c.489+1G>T, p.Glu585X (c.1753G>T), p.Phe508del, p.Gly542X (c.1624G>T), p.Gly551Asp, p.Asn1303Lys (c.3909C>G), p.Arg553X (c.1657C>T), p.Val520Phe (c.1558G>T), or p.Trp1282X (c.3846G>A). Mutation legacy names can be found in the Cystic Fibrosis Mutation Database (http://www.genet.sickkids.on.ca).

### Genotyping

Linkage disequilibrium between SNPs was assessed using Haploview (http://www.broad.mit.edu/mpg/haploview) [66]. DNA extracted from either whole blood or transformed lymphocyte cell lines was hybridized to the Illumina Infinium 610-Quad SNP array platform for whole genome genotyping at the McGill University and Génome Québec Innovation Centre. Genotyping was performed and stringent quality control measures were employed simultaneously in both cohorts, and the quality of SNP calls was deemed to be very high (0.004% discordance between replicate samples). SNPs were excluded from analysis in all cohorts if the call rate was <90%, if the minor allele frequency was <1%, or if the Mendelian error rate was >1%. For family-based studies, any marker displaying non-Mendelian inheritance was dropped from analysis for any family with the error. A detailed description of additional quality control measures can be found in Wright, *et al* [34].

### Statistical analysis

For the primary study, family-based association testing of SNPs and haplotypes was performed using the PBAT module [67] implemented within the Golden Helix HelixTree software package (Golden Helix, Inc. Bozeman, MT, USA; http://www.goldenhelix.com). A sliding-window approach was employed to test the transmission of haplotypes composed of three adjacent SNPs (frequency >1%). A 2 Mb region was selected for haplotype testing as the number of possible unique haplotypes would be nearly equal to the number of SNPs tested in the initial analysis. Therefore, to be considered significant, a haplotype would have to reach the same Bonferroni-corrected threshold (or higher) that was set for SNPs in the initial analysis. An additive genetic model was applied under a null hypothesis of linkage and no association, and standard phenotypic residuals were used as offsets to increase the power of the test statistic. Bonferroni correction was applied by multiplying nominal *P* values by the total number of SNPs or haplotypes tested. The SNP association plot was generated using LocusZoom 1.0 (http://csg.sph.umich.edu/locuszoom).

For case/control analyses, haplotypes were derived in the primary and replication populations using an expectation-maximization (EM) algorithm implemented in Golden Helix. Statistical analysis was performed in Stata10 (StataCorp, College Station, TX, USA). Comparison of MI status to the number of copies of haplotypes was performed using Fisher's exact test (using only subjects with haplotypes that could be determined with 100% posterior probability). Odds ratios comparing the odds of MI in subjects with 0, 1 or 2 copies of the chr8 haplotype, thus assuming an additive model, were estimated using logistic regression. For subjects with more than one haplotype assignment, EM probability estimates were used to weight haplotypes. For TSS subjects, parental and sibling genotypes were utilized when possible to resolve phase. For studies including siblings (TSS and CGS), empiric standard errors account for the possibility of sib-pair correlation [68].

### Mouse studies

Mice heterozygous for a *Cftr* null allele, B6.129P2-*Cftr^tm1Unc^* [17] or for the *Cftr* missense allele p.Arg117His, B6.129S6-*Cftr^tm2Uth^* [44], and either homozygous or heterozygous for a null allele of *Msra* [51] were generated as breeders to produce CF mice carrying the three genotypes of *Msra* (+/+, +/−, and −/−) used in this study. Mice were housed at constant temperature (22°C) on a 12 hour light/dark cycle. Cages were checked daily for births and for monitoring health and survival of animals to 40 days. Mice were weaned at 21 days of age onto an enriched diet (9F Sterilizable Rodent Diet 7960, Harlan Teklad, Madison, WI) and provided water *ad libitum*. Mice were of mixed background, but predominantly C57BL/6J (>87%; crossed a minimum of two generations to C57BL/6J and some animals up to ten generations).

Genotyping was carried out on 7 to 10-day old animals and if death occurred before this point, genotypes were determined from carcasses. *Cftr* genotyping was performed as previously described [44]. *Msra* genotyping was carried out using two primer sets to generate a 579-bp product for the wild-type allele (forward 5′-GTGTGAGAATAAACAGATGTTCTATGC-3′ and reverse 5′-GGGTTGAGTACACTCCTTTCA-3′) or a 320-bp product for the mutant null allele (forward 5′-AAAGCGCCTCCCTACCCG-3′ and reverse 5′-ACTGTGCCCAGTTTAGTCCGTG-3′). Samples were amplified by an initial denaturation for 5 min at 95°C followed by 35 cycles of 95°C for 30 sec, 59°C for 30 sec, and 72°C for 30 sec. PCR products were fractionated on a 1% agarose gel. Kaplan-Meier survival curves were plotted and differences in survival were analyzed by the non-parametric test of trends and log-rank test of equality using Stata10.

For histology, freshly harvested tissues were fixed in 10% formalin. Tissue was embedded in paraffin, sectioned at 5 µm thickness on a microtome, and mounted on glass slides for microscopy. Deparaffinized slides were stained with Alcian blue and periodic acid Schiff (PAS) stains, then counterstained with acidified Harris hematoxylin. Comparison of goblet cell proportions between groups was performed using the non-parametric Mann-Whitney test.

## Supporting Information

Figure S1 Haplotype association in *MSRA* region. Three-SNP haplotypes were tested for association within a 2 Mb window flanking the most highly associated SNP from the initial analysis (rs614197, bold). *P* values are plotted in log scale versus physical location in Mb. Each dot represents the mean position of the three consecutive SNPs comprising a given haplotype. Two overlapping haplotypes were significantly associated with protection against MI after Bonferroni correction for 2,890 observed haplotypes (dashed line; *P*<1.73×10^−5^) and a third haplotype just below this threshold was associated with risk for MI. The two protective haplotypes are localized within intron 3 of *MSRA* and the “risk” haplotype is 5′ to *MSRA*, as shown in Figure 2. The SNPs comprising these haplotypes (larger dots) are labeled and underlining indicates SNPs in common between haplotypes. Genes, exon positions, and direction of transcription are denoted below plot.
(JPG)
