# Supplementary figures and images for: Variation in MSRA Modifies Risk of Neonatal Intestinal Obstruction in Cystic Fibrosis
Source: PLoS Genet. 2012 Mar 15;8(3):e1002580. doi: 10.1371/journal.pgen.1002580 (PMC3305406; doi:10.1371/journal.pgen.1002580)

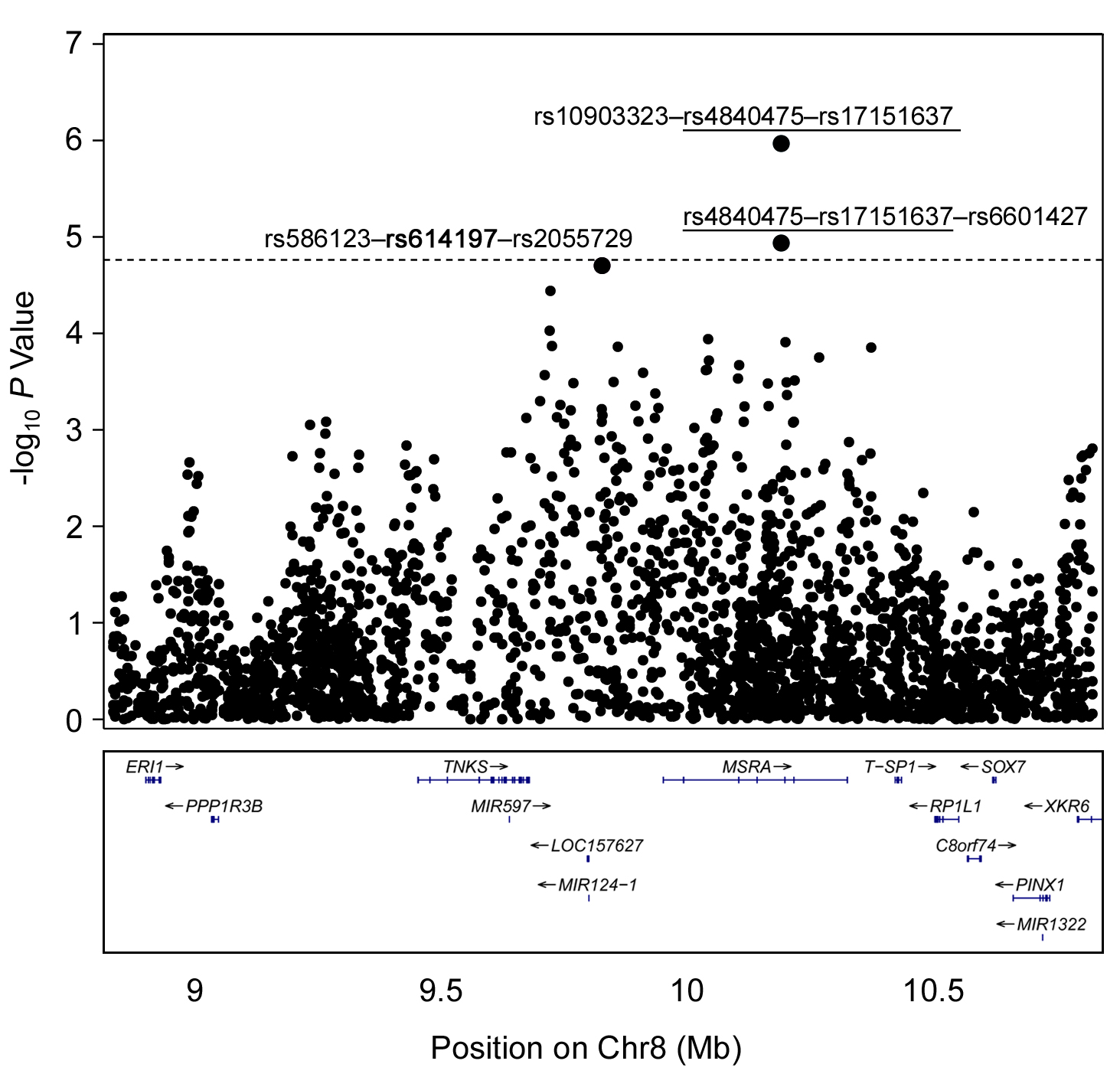

Supplement: Figure S1 — Haplotype association in MSRA region. Three-SNP haplotypes were tested for association within a 2 Mb window flanking the most highly associated SNP from the initial analysis (rs614197, bold). P values are plotted in log scale versus physical location in Mb. Each dot represents the mean position of the three consecutive SNPs comprising a given haplotype. Two overlapping haplotypes were significantly associated with protection against MI after Bonferroni correction for 2,890 observed haplotypes (dashed line; P<1.73×10^−5^) and a third haplotype just below this threshold was associated with risk for MI. The two protective haplotypes are localized within intron 3 of MSRA and the “risk” haplotype is 5′ to MSRA, as shown in Figure 2. The SNPs comprising these haplotypes (larger dots) are labeled and underlining indicates SNPs in common between haplotypes. Genes, exon positions, and direction of transcription are denoted below plot. (JPG) [file pgen.1002580.s001.jpg]
